# Immediate gain is long-term loss: Are there foresighted decision makers in the Iowa Gambling Task?

**DOI:** 10.1186/1744-9081-4-13

**Published:** 2008-03-19

**Authors:** Yao-Chu Chiu, Ching-Hung Lin, Jong-Tsun Huang, Shuyeu Lin, Po-Lei Lee, Jen-Chuen Hsieh

**Affiliations:** 1 Department of Psychology, Soochow University, Taipei, Taiwan; 2 Institute of Neuroscience, School of Life Science, National Yang-Ming University, Taipei, Taiwan; 3 Laboratory of Integrated Brain Research, Department of Medical Research & Education, Taipei Veterans General Hospital, Taipei, Taiwan; 4 Institute of Neural and Cognitive Sciences, China Medical University & Hospital, Taichung, Taiwan; 5 Department of Psychology, National Taiwan University, Taipei, Taiwan; 6 Department of Business Administration, Minghsin University of Science and Technology, Hsinchu, Taiwan; 7 Department of Electrical Engineering, National Central University, Taoyuan, Taiwan; 8 Institute of Brain Science, School of Medicine, National Yang-Ming University, Taipei, Taiwan

## Abstract

**Background:**

The Somatic Marker Hypothesis suggests that normal subjects are "foreseeable" and ventromedial prefrontal patients are "myopic" in making decisions, as the behavior shown in the Iowa Gambling Task. The present study questions previous findings because of the existing confounding between long-term outcome (expected value, EV) and gain-loss frequency variables in the Iowa Gambling Task (IGT). A newly and symmetrically designed gamble, namely the Soochow Gambling Task (SGT), with a high-contrast EV between bad (A, B) and good (C, D) decks, is conducted to clarify the issue about IGT confounding. Based on the prediction of EV (a basic assumption of IGT), participants should prefer to choose good decks C and D rather than bad decks A and B in SGT. In contrast, according to the prediction of gain-loss frequency, subjects should prefer the decks A and B because they possessed relatively the high-frequency gain.

**Methods:**

The present experiment was performed by 48 participants (24 males and 24 females). Most subjects are college students recruited from different schools. Each subject played the computer version SGT first and completed a questionnaire for identifying their final preference. The IGT experimental procedure was mostly followed to assure a similar condition of decision uncertainty.

**Results:**

The SGT experiment demonstrated that the prediction of gain-loss frequency is confirmed. Most subjects preferred to choose the bad decks A and B than good decks C and D. The learning curve and questionnaire data indicate that subjects can not "hunch" the EV throughout the game. Further analysis of the effect of previous choice demonstrated that immediate gain increases the probability to stay at the same deck.

**Conclusion:**

SGT provides a balanced structure to clarify the confounding inside IGT and demonstrates that gain-loss frequency rather than EV guides decision makers in these high-ambiguity gambles. Additionally, the choice behavior is mostly following the "gain-stay, lose-randomize" strategy to cope with the uncertain situation. As demonstrated in SGT, immediate gain can bring about a long-term loss under uncertainty. This empirical result may explain some shortsighted behaviors in real life.

## Background

Studies in behavioral decision-making and affective neuroscience have found that typical decision makers are frequently "myopic" [1-5] to long-term outcome (expected value, EV) [6]. Conversely, Damasio [7] and Bechara et al. [8,9] proposed the Somatic Marker Hypothesis and conducted the Iowa Gambling Task (IGT) to test whether ventromedial prefrontal patients are shortsighted in terms of the future and long-term outcome, and whether typical decision makers can predict or foresee the future. For comparison, the notion of shortsighted vs. foresighted is adopted in this study. A marked difference exists between shortsightedness and foresight. The IGT was the central test for a verification of the Somatic Marker Hypothesis. Some studies have attempted to replicate or modify the protocol used in the IGT [10-12]; whereas others have proposed that the hypothesis was theoretically inadequate [1,5,13-16]. Lin et al. [17] pointed out that there are increasing number of studies [18-28] to demonstrate a contradictory phenomenon, namely, the "prominent deck B phenomenon" [29]. The phenomenon showed that normal decision makers can not prevent their preference to "bad" (EV) deck B in the standard version of IGT (or, due to the effect of "gain-loss frequency"). Recently, Bechara (Sevy et al.) [30,31] also revealed a "prominent deck B phenomenon". In their study, normal subjects preferred the bad deck B rather than the other three decks. Furthermore, the chosen number of deck B (31) in Sevy et al. study [30] is almost a double of Bechara et al. data in 1994 (about 17) [8]. On the other hand, Lin et al. [29] analyzed the existing experimental results adopting simple version of IGT and suggest that the "prominent deck B phenomenon" may be due to a confounding from gain-loss frequency. Additionally, Chiu and Lin [32] utilize a modified version of IGT to demonstrate that subjects' preference to deck C is also due to gain-loss frequency, not EV. A fundamental structural flaw exists in a failure of orthogonal separation between EV and gain-loss frequency (or, frequency of punishments and rewards). This limitation has not been properly evaluated in IGT literature. The study tries to explore the implications of this flaw.

Damasio's [7] Somatic Marker Hypothesis proposes that normal decision-making is often assisted by somatic markers. Ventromedial prefrontal patients are influenced largely by immediate reinforcement and are insensitive to, or cannot see, future consequences due to a lack of past affective experiences. Normal subjects with intact somatic markers benefit from repeated exposure to punishments and rewards when performing tasks and are cognizant of future outcome. Somatic states serve as a neural expression biasing the brain process when evaluating the badness or goodness of each decision. According to Damasio [7]:

"*... the brains of the normal subjects were gradually learning to predict a bad outcome, and were signaling the relative badness of the particular deck before the actual card-turning*." (Damasio, 1994, p 220).

In short, the Somatic Marker Hypothesis suggests that somatic markers, which are processed implicitly, can facilitate decision makers in making advantageous decisions [9] and guiding explicit decision making [33,34].

The most compelling empirical evidence for supporting the Somatic Marker Hypothesis is found in the IGT [8]. Over the past decade, the IGT has been widely employed as a neuropsychological research instrument for investigating affective and executive function. At least 100 scientific studies have utilized the IGT to investigate a diverse set of neurological and psychiatric populations [35].

In the IGT, card decks A and B were designated "bad" decks with low EV ($ -250), and C and D were "good" decks with high EV ($ +250) in average 10 trials. Subjects and ventromedial prefrontal patients selected "bad" decks during the first 30 card-turning trials (out of 100) [7,36]; however, normal subjects gradually shifted to "good" decks and avoided the "bad" decks [8,9,36-39]. However, a careful examination of the IGT reveals a critical confounding between EV and gain-loss frequency. On average, for each 10-card unit, deck A contains 5 gains and 5 losses, and deck B contains 9 gains and 1 loss. On the other hand, deck C contains 6.25 gains, 2.5 standoffs and 1.25 losses, and D contains 9 gains and 1 loss. Altogether the bad decks (A and B) contain 14 gains and 6 losses, whereas good decks (C and D) contain 15.25 gains, 2.5 standoffs, and only 2.25 losses. Both good and bad decks have a similar number of gains, whereas the good decks have significantly fewer losses (see Tables 1 and 2). Therefore, it is not clear whether subjects' choices of good decks in the IGT were driven by improved EV or gain-loss frequency. This study attempts to generate a symmetrical and fair experiment to demonstrate the relative guiding power of gain-loss frequency and EV under uncertainty, specifically to identify which factor most comprehensively dominates normal subject preferences. To differentiate between the relative contributions of EV and gain-loss frequency, this study applies a new task, namely, the Soochow Gambling Task (SGT) [40,41] (see Table 1).

**Table 1:** The immediate net value of each trial and gain-loss structure in the original IGT and SGT

***IGT Serial Numbers***	**A**	**B**	**C**	**D**	***SGT***	**A**	**B**	**C**	**D**
***1***	100	100	50	50	***1***	200	100	**-200**	**-100**
***2***	100	100	50	50	***2***	200	100	**-200**	**-100**
***3***	**-50**	100	0	50	***3***	200	100	**-200**	**-100**
***4***	100	100	50	50	***4***	200	100	**-200**	**-100**
***5***	**-200**	100	0	50	***5***	**-1050**	**-650**	1050	650
***6***	100	100	50	50	***6***	200	100	**-200**	**-100**
***7***	**-100**	100	0	50	***7***	200	100	**-200**	**-100**
***8***	100	100	50	50	***8***	200	100	**-200**	**-100**
***9***	**-150**	**-1150**	0	50	***9***	200	100	**-200**	**-100**
***10***	**-250**	100	0	**-200**	***10***	**-1050**	**-650**	1050	650

***11***	100	100	50	50	***11***	200	100	**-200**	**-100**
***12***	**-250**	100	25	50	***12***	200	100	**-200**	**-100**
***13***	100	100	**-25**	50	***13***	200	100	**-200**	**-100**
***14***	**-150**	**-1150**	50	50	***14***	200	100	**-200**	**-100**
***15***	**-100**	100	50	50	***15***	**-1050**	**-650**	1050	650
***16***	100	100	50	50	***16***	200	100	**-200**	**-100**
***17***	**-200**	100	25	50	***17***	200	100	**-200**	**-100**
***18***	**-50**	100	**-25**	50	***18***	200	100	**-200**	**-100**
***19***	100	100	50	50	***19***	200	100	**-200**	**-100**
***20***	100	100	0	**-200**	***20***	**-1050**	**-650**	1050	650

***21***	100	**-1150**	50	50	***21***	200	100	**-200**	**-100**
***22***	**-200**	100	50	50	***22***	200	100	**-200**	**-100**
***23***	100	100	50	50	***23***	200	100	**-200**	**-100**
***24***	**-250**	100	0	50	***24***	200	100	**-200**	**-100**
***25***	100	100	25	50	***25***	**-1050**	**-650**	1050	650
***26***	**-100**	100	0	50	***26***	200	100	**-200**	**-100**
***27***	**-150**	100	50	50	***27***	200	100	**-200**	**-100**
***28***	**-50**	100	50	50	***28***	200	100	**-200**	**-100**
***29***	100	100	**-25**	**-200**	***29***	200	100	**-200**	**-100**
***30***	100	100	0	50	***30***	**-1050**	**-650**	1050	650

***31***	**-250**	100	50	50	***31***	200	100	**-200**	**-100**
***32***	**-100**	**-1150**	50	50	***32***	200	100	**-200**	**-100**
***33***	**-150**	100	50	50	***33***	200	100	**-200**	**-100**
***34***	100	100	25	50	***34***	200	100	**-200**	**-100**
***35***	100	100	25	**-200**	***35***	**-1050**	**-650**	1050	650
***36***	100	100	50	50	***36***	200	100	**-200**	**-100**
***37***	**-50**	100	**-25**	50	***37***	200	100	**-200**	**-100**
***38***	**-200**	100	50	50	***38***	200	100	**-200**	**-100**
***39***	100	100	0	50	***39***	200	100	**-200**	**-100**
***40***	100	100	**-25**	50	***40***	**-1050**	**-650**	1050	650

***Final Outcomes***	**-1000 ($)**	**-1000 ($)**	**+1000 ($)**	**+1000 ($)**	***Final Outcomes***	**-2000 ($)**	**-2000 ($)**	**+2000 ($)**	**+2000 ($)**

*Note*. In these gambling tasks, the internal gamble structure of IGT and SGT and the trial to end the game is unknown to the subjects. The subjects are asked to minimize monetary expenditure and maximize their winnings by choosing one card from the four decks in each trial. In the table, negative values are marked with a bold font size. (Left part: IGT) Deck A contains the relative high-frequency loss, while decks B, C, and D contain the high-frequency gain (net value within each trial). Decks A and B have negative net value (namely, the EV), -250($) over an average of ten trials; moreover, C and D have a positive net value of +250($) over an average of ten trials. (Right part: SGT) Five cumulative trials are repeated for each deck in the Soochow Gambling Task, decks A and B have high-frequency gain, while decks C and D exhibit a reversed gain-loss pattern. The task enlarges the difference between positive and negative EVs to make the difference more noticeable than in the Iowa Gambling Task. While playing this game, subjects only experienced a gain or a loss during each trial, and there was no reciprocal gain-loss within individual trials.

**Table 2:** A comparison of gamble structures between the Iowa Gambling Task and Soochow Gambling Task

**Iowa Gambling Task**	**Gain-Loss Frequency (per 10 cards)**	**EV (per 10 cards)**	**Choice Prediction Based on Gain-Loss Frequency**	**Choice Prediction Based on EV**
A (Bad)	5 G 5 L	-$250		
B (Bad)	9 G 1 L	-$250	B	
C (Good)	6.25 G 1.25 L 2.5 S	+$250	C	C
D (Good)	9 G 1 L	+$250	D	D

Soochow Gambling Task *	**(per 5 cards)**	**(per 5 cards)**		

A (Bad)	4 G 1 L	-$250	A	
B (Bad)	4 G 1 L	-$250	B	
C (Good)	1 G 4 L	+$250		C
D (Good)	1 G 4 L	+$250		D

(G: gain, L: loss, S: standoff).

* The deck structure for the Soochow Gambling Task is as follows: A, four consecutive gains of $200 followed by a loss of $1,050; B, four consecutive gains of $100 followed by a loss of $650; C, four consecutive losses of $200 followed by a gain of $1,050; D, four consecutive losses of $100 followed by a gain of $650. The experiment was using token money denominated in New Taiwan Dollars. For ease of comparison, the amount of money was nearly equated to U.S. currency in IGT.

## Method

A two-phase study was designed to explore learning process and final preferences during decision making. Four card decks, randomized into 24 arrangements (e.g., ABCD, BCDA, CDAB, etc.), were presented on a computer screen. Forty-eight college students and adults (24 males, 24 females; age: 18–30 years, mean age: 20.71 years) participated in this study. Each subject undertook one set of card arrangement to balance the position effect. After completing the computer game, subjects answered a questionnaire that gauged their memories of task characteristics and their deck preferences.

The SGT contains four decks, each containing 5 card-turning trials as a unit, for a total of 100 trials. The "bad" decks (defined by EV, as in IGT) in the SGT have worse EV ($ -250) and better gain-loss frequency (4 gains and 1 loss), whereas the "good" decks have better EV ($ +250) and worse gain-loss frequency (1 gain and 4 losses). Thus, in the SGT, the operation of gain-loss frequency and EV variables will predict different choice patterns. If decision-making is guided by EV, subjects will prefer the good decks (C and D) over the bad decks (A and B). Conversely, when participant choice behavior is controlled by immediate gain-loss, the perceived gain will keep subjects on the same deck. The deck with the highest number of gains will increase the probability of being chosen. Table 2 summarizes a comparison of the task structures and predictions between the IGT and the SGT.

The SGT adopted an experimental procedure similar to that used in the IGT to assure that subjects perform the task under uncertain conditions. The probability structures of gain-loss frequency were unknown to subjects in both tasks (IGT and SGT). Subjects were also ignorant of the time limitation in the experiment. Both tasks then are uncertain in the traditional sense.

This study adopted the subject instructions employed by Bechara et al. [36]. The key points of the instructions are as follows:

"*The goal of the game is to win as much money as possible and, if you find yourself unable to win, make sure you avoid losing money as much as possible. I won't tell you for how long the game will continue. You must keep on playing until the computer stops. It is important to know that the colors of the cards are irrelevant in this game. The computer does not make you lose money at random. However, there is no way for you to figure out when the computer will make you lose. All I can say is that you may find yourself losing money on all decks, but some decks will make lose more than others. You can win if you stay away from the worst decks ....*" (Bechara et al., 1999, p. 5474).

## Results

**Figure 1:**
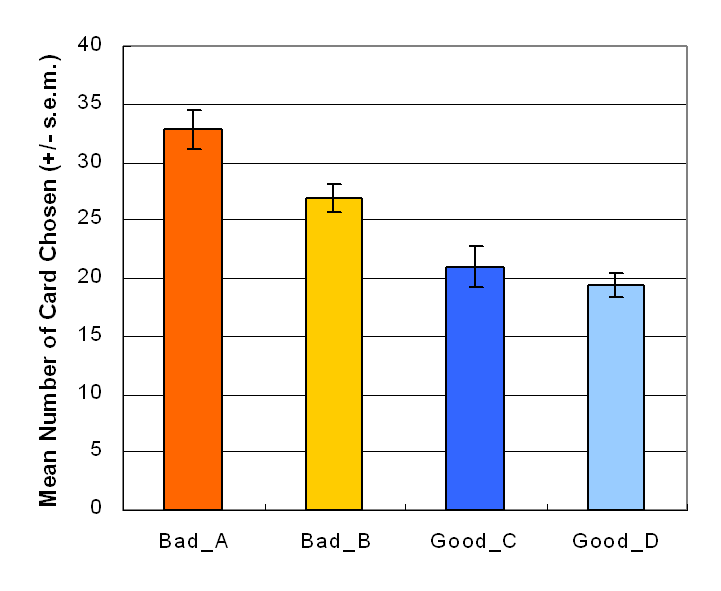
**Mean number of cards chosen**. The results of two factors (repeated measurement) ANOVA (Gain-loss frequencies (gain vs. loss) × Values (± 200 vs. ± 100)) showed a significant effect of gain-loss frequency. Subjects selected more cards from decks A and B than decks C and D (*F *(1, 47) = 26.41, *p *< .01). The value effect with of the paired t-test is significant (*t *(47) = 2.60, *p *< .05) under high-frequency gain (+200 vs. +100), but not significant (*t *(47) = 0.72, *p *= .48) under high-frequency loss (-200 vs. -100). None of the interaction effects are statistically significant (*F *(1, 47) = 1.88, *p *= .18).

Experimental results showed that subjects preferred the bad decks (A and B) to the good decks (C and D) (Figure 1). This finding supports the effect of prediction-based gain-loss frequency and is contrary to that of EV.

**Figure 2:**
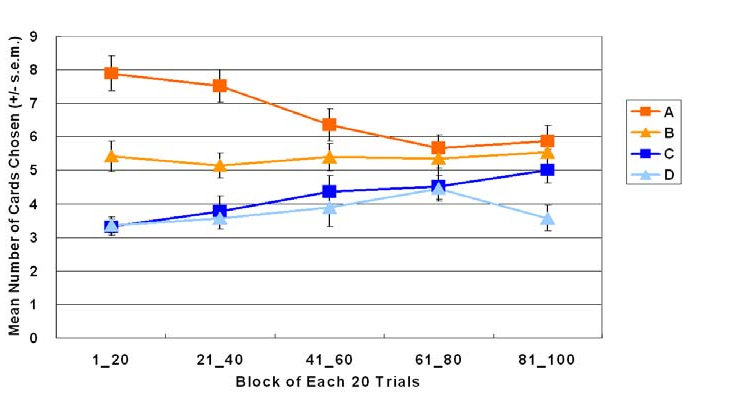
**Mean number of cards chosen in blocks**. 100 card selection trials were grouped into five blocks, each comprising 20 trials. The three factor (repeated measurement) ANOVA (Gain-loss frequencies (gain vs. loss) × Values (± 200 vs. ± 100) × Blocks (1 to 5)) indicated a significant main effect for gain-loss frequencies (*F *(1, 47) = 29.44, *p *< .01) and values (*F *(1, 47) = 9.02, *p *< .01), but not for blocks (*F *(1, 47) = 0.00, *p *= 1.00). Furthermore, significant interactions existed between gain-loss frequencies and blocks (*F *(4, 44) = 3.03, *p *< .05) as well as three factors (*F *(4, 44) = 5.19, *p *< .01); but non-significant interactions existed between gain-loss frequencies and values (*F *(1, 47) = 1.90, *p *= .18); values and blocks (*F *(4, 44) = 0.99, *p *= .43). These results indicate a clear preference for the pooled decks A and B ("bad" decks) over the pooled decks C and D ("good" decks) from the beginning. Subjects seem to be guided by gain-loss frequencies and appear sensitive to the gain-loss structure gradually. No cross-over or significant learning curve exists for the high-frequency gain (A, B) and high-frequency loss (C, D) decks under this condition (100 trials) in the Soochow Gambling Task.

Moreover, the average number of times subjects chose bad decks was higher than that for good decks throughout the entire experiment (Figure 2). Significant interaction was found between gain-loss frequency and blocks. However, no single main effect of blocks was observed. Subjects gradually adjusted their selection pattern to the chance level of four choices. The absence of a crossover or lack of learning curve indicates that gain-loss frequency was dominant over EV when subjects were choosing decks.

**Figure 3:**
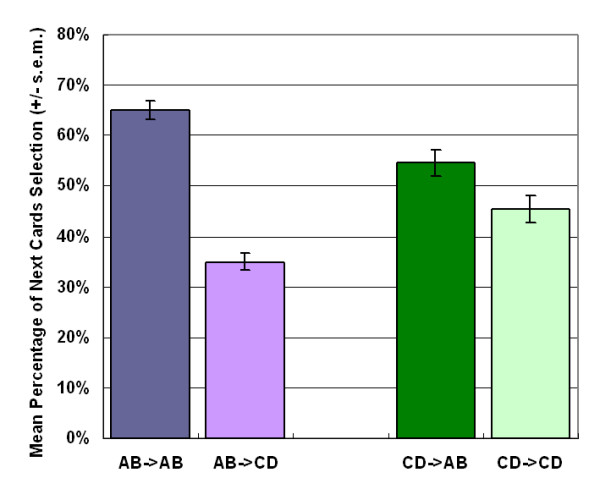
**Mean probability of shift and stay in the continuing choice**. After choosing the (bad) decks A or B, 65% of participants remain on these two decks, with only 35% shifting to the (good) decks C and D (*t *(47) = 8.60, *p *< .01). On the other hand, when subjects choose decks C and D, the probabilities of them shifting or staying in their next selection was a roughly 50/50 probability of selecting deck A and B versus C and D (*t *(47) = 1.71, *p *= .09).

Furthermore, this investigation also analyzes the pattern of continuing choices after the subject chose AB or CD (Figure 3). The experimental results showed that subjects tend to remain on the same type of decks while choosing A or B. Nevertheless, no significant differences are found for the continuing choices between the same or the different types of decks while they were choosing C or D.

**Figure 4:**
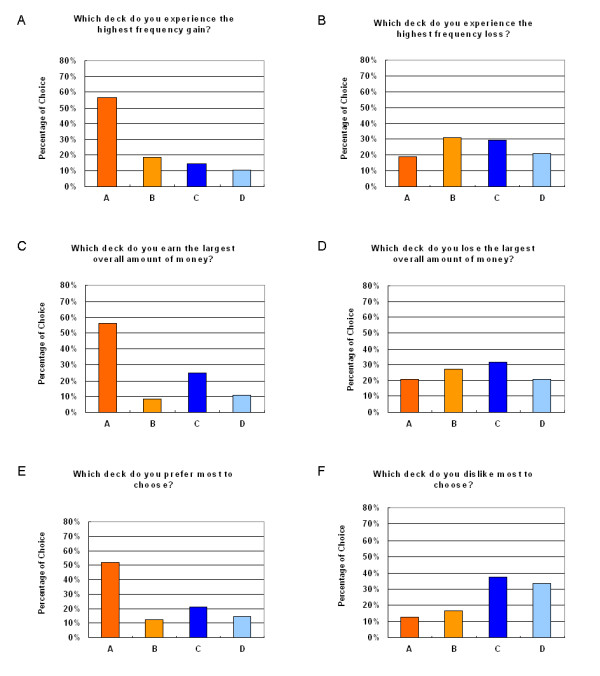
**Subject memory assessment in the Soochow Gambling Task**. Forty-eight subjects were required to report their behavior and preferences after completion of the game. (4A) Most of the sample (36 subjects) had vivid impressions for high-frequency gain decks (A+B) (*x*^2 ^(1) = 12.00, *p *< .01), (4B) but not high-frequency loss (C+D) (*x*^2 ^(1) = 0.00, *p *= 1.00). (4C) Additionally, most subjects (31 subjects recognized decks A and B) possessed a clear (but wrong) image for the overall monetary gain (*x*^2 ^(1) = 4.08, *p *< .05); (4D) but a blurred image for overall monetary loss (*x*^2 ^(1) = .08, *p *= .77). (4E) After completing the game, subjects erroneously equated the high-frequency gains as the overall advantage. Thirty one out of forty eight subjects indicated the favorable choice to decks A and B rather than C and D (*x*^2 ^(1) = 4.08, *p *< .05). (4F) Most unfavorable choice they had memorized were decks C and D which possessed the high-frequency loss and positive expected value (*x*^2 ^(1) = 8.33, *p *< .01).

Analysis of the questionnaire results indicated that subjects correctly recalled which decks they chose that had the highest gains (Figure 4A); but subjects have a chance-level recollection on which decks they chose that had the highest losses (Figure 4B). Conversely, subjects incorrectly recalled which decks were in use when they won the largest overall amount of money (Figure 4C); but subjects have a chance-level to recall which decks were chosen when they lost the largest overall amount of money (Figure 4D). Preference patterns of conscious recollection were consistent with the choice pattern due to the effect of gain-loss frequency (Figures 4E and 4F).

## Discussion

Subjects may apply an implicit strategy to cope with the uncertain game, therefore they favored high-frequency gains over high-frequency losses in the experiment. This "gain-stay, lose-randomize" strategy (Figure 3) [42] has been observed in human and animal appetitive and avoidance experiments in which human or animal encounter reward or punishment [42-48]. These pioneer behavior studies with the concurrent schedules of reinforcement have displayed the frequency effect for choice pattern [45-49]. Additionally, these concepts have also been applied to examine the behavioral model of neuropsychological deficit [50,51].

The SGT possessed the variant frequency, magnitude and delay of reinforcement/punishment depending on each subject's choice. Most part of these set-ups may be similar to the consideration of the traditional behavior-analysis studies [45-49,52], therefore, the gain-loss frequency can successfully serve as a predictor for choice behavior under these uncertain situations.

The effect of gain-loss frequency is not an isolated finding in similar settings. A reexamination of the bad decks (A and B) in the original IGT [8] indicated that bad deck B (9 gains and 1 loss) was also chosen more frequently than deck A (5 gains and 5 losses). Other studies obtained similar findings but did not explore them further [18-28,33,34]. Furthermore, some research groups even showed that normal subjects chose the disadvantageous deck B more frequently than the advantageous deck C or D [18-28]. Dunn et al. [14] sampled 38 IGT related studies to demonstrate that only five studies [18,19,23,25,53] utilized the "four-deck format" to display their findings (i.e., the number of card turnings for each deck over a total of 100 trials being shown separately). These studies all demonstrated that deck B was chosen more frequently than deck A. It is worth noting that four out of five studies [18,19,23,25,53] also demonstrated that deck B was chosen more than deck C or D.

Furthermore, in Peters and Slovic study [54], the modified IGT study also demonstrated that deck B ($ -250) and D ($ +250) possessed the inversed expected values, but with nearly equal attraction to subjects. This may imply that the expected value does not guide decision makers to approach the beneficial choice in these dynamic games. On the other hand, in the modified IGT, deck C contained high-frequency gain (8 out of 10 trials) over deck D (5 out of 10 trials), but with nearly equal expected value ($ +300 for C vs. $ +250 for D). However, subjects preferred deck C rather than deck D significantly. The gain-loss frequency seems to be more reasonable than EV in explaining these observations. Ahn et al. [55] confirmed the present finding by comparing the decision learning models for IGT and SGT respectively.

Questionnaire data in this study also indicate a novel phenomenon, namely, the "money account illusion". Subjects ignored the EV dimension and miscounted the money amount in terms of the strength of frequency, specifically, frequently receiving gains will leave an overall impression of large accumulated monetary outcome for a deck than when gains are infrequently received.

In the IGT, normal subjects shifted their choices to good decks during the latter part of the game, such that a learning curve was evident [7-9,36,37,39]. We propose that this shift is not due to better EV but rather an effect resulting from more frequent gains than losses. The Somatic Marker Hypothesis stresses that somatic markers (or peripheral feedback) predispose normal subjects to behave in accordance with perceived future consequences over the long run. This study demonstrated that even subjects with intact somatic markers cannot behave accordingly to a search for EV in the SGT. Immediate reinforcement will override EV in the SGT. Decision makers may have been guided by immediate gain and, as such, their behavioral results are consistent with the prediction of gain-loss frequency. Based on the present observation, three possible interpretations exist for SMH: 1) somatic marker system may guide decision making behavior via rough-estimation processing (gain-loss frequency), not a precise calculation (EV: probability × value); 2) somatic marker system may only contribute to generating subjective feelings (consciousness), and may not be immediately related to decision guidance; 3) the operation of the somatic marker may be involved in gathering the long-term memory, but may not globally direct choice behavior in situations of high uncertainty.

The Somatic Marker Hypothesis also posits that somatic markers guide advantageous behavior in a non-conscious manner. However, questionnaire results suggest further that subjects can have a clear knowledge of gain and loss frequency by the end of the game. This perception eventually determines their choice patterns. A similar finding was obtained by Maia and McClelland [34], demonstrating a possible "conscious" knowledge of EV in the IGT.

In the SGT, normal subjects were stuck with the influence of gain-loss frequency without shifting to EV throughout the entire session. If normal subjects cannot resist the influence of gain-loss frequency, ventromedial prefrontal patients would have increased vulnerability to the effect of immediate gains and losses. This prediction seems to be in line with disinhibition theory that suggests that socially dysfunctional patients with prefrontal damage have difficulty avoiding a punishment-associated stimulus when that stimulus was previously associated with a reward [13,15,16,56]. If this is true, then both normal subjects and ventromedial prefrontal patients will not be responsive to the long-term dimension.

Supposing this is the case, the facilitative effect of somatic markers did not induce a learning effect or an advantageous shifting behavior in the long run in the SGT, as consistently proposed by IGT researchers. Careful analysis of experimental results obtained in this study identified a subordinate phenomenon in that deck A was chosen more often than deck B, despite both decks possessing the same gain-loss frequency. The immediate value of gain and loss can also alter slightly decision-makers' choices and is worthy of further exploration.

EV and gain-loss frequency were seriously confounded in the administration of the IGT. Selection of good decks by normal subjects cannot only be attributed to the effect of EV, but must also be explained in terms of gain-loss frequency. The Somatic Marker Hypothesis was further reinforced by adopting the principal findings from the IGT experiment as important supporting data, i.e., somatic markers predispose normal subjects to search for EV in the long run. This study explored separately the relative contribution of EV and gain-loss frequency in the SGT, a modified gambling task. Experimental results indicated that immediate reinforcement overrides the effect of EV. Crone et al. [57] also identified a local preference for gain-loss frequency; however, EV dominated gain-loss frequency in their modified IGT. In contrast to the predictions based on Somatic Marker Hypothesis, experimental data in this study indicate that normal subjects were primarily guided by the effect of gain-loss frequency rather than EV. This finding, although differing from the simple explanation provided by the Somatic Marker Hypothesis, is consistent with the view of behavioral [1-5] and affective [5,20,58-62] decision literature, indicating that normal individuals are often short-sighted when making decisions in stock market or real life [41,63,64].

## Conclusion

A serious confounding effect in IGT was demonstrated by the "prominent deck B phenomenon". This recently-discovered phenomenon may imply that gain-loss frequency rather than EV dominates the choice behavior of normal decision makers under uncertainty. In the original IGT, these two factors were seriously confounded, and thus this study proposed a modified task SGT to clarify the relative contribution of these two factors. The experimental results indicated that subject selection patterns were mostly predicted based on gain-loss frequency. Immediate gain may increase the probability of continuing to stick on the chosen deck, which is consistent with most behavioral-analysis and decision literatures. The observations of this study indicate that EV is not predictive for choice behaviors as suggested by IGT. It seems that immediate gain eventually resulted in a long-term loss. The basic assumption of IGT is thus no longer plausible to assure the foresighted decision makers in the Iowa Gambling Task.

## Abbreviations

EV: Expected Value; IGT: Iowa Gambling Task; SGT: Soochow Gambling Task; SMH: Somatic Marker Hypothesis

## Competing interests

The authors certify that the information listed above is complete to the best of our original research. The authors declare that they have no competing interests.

## Authors' contributions

This design of Soochow Gambling Task was constructed by YC and CH, both have made equal contributions to thought, data interpretation, drafting the key concept, and revising it critically. JT provided several times of critical review to organize the whole picture for this work and revised this article significantly. PL worked on the computerization of task, consulting of data analysis. SY and JC participated in some discussion and provided some valuable viewpoints to improve the meaning of data and readability. All authors gave final approval of the version to be published.
